# Ancient Jewel-Pointed Tools

**Published:** 1893-01

**Authors:** 


ANCIENT JEWEL-POINTED TOOLS.—A year's study at Gizeh has convinced Mr. Flinders Petrie that the Egyptian stone-workers of 4,000 years ago had a surprising acquaintance with what have been considered modern tools. Among the many tools used by the pyramid-builders were both solid and tubular drills and straight and circular saws. The drills, like those of to-day, were set with jewels, (probably corundum, as the diamond was very scarce,) and even lathe tools had such cutting edges.

So remarkable was the quality of the tubular drills and the skill of the workmen that the cutting marks in hard granite give no indication of wear of the tool, while a cut of a-tenth of an inch was made in the hardest rock at each revolution, and a hole through both the hardest and softest material was bored perfectly smooth and uniform throughout. Of the material and method of making the tools nothing is known.

